# Is non-invasive blood pressure comparably accurate to the invasive method in cats?

**DOI:** 10.18849/ve.v11i2.735

**Published:** 2026-06-24

**Authors:** Fiona Douglas

**Keywords:** BLOOD PRESSURE, DIAGNOSTIC TEST EVALUATION, DOPPLER, FELINE, HYPERTENSION, OSCILLOMETRIC

## Abstract

**PICO question:**

In cats, is blood pressure measurement using non-invasive methods (Doppler or oscillometric) comparable to invasive arterial blood pressure measurement (direct arterial telemetry)?

**Clinical bottom line:**

**Category of research:**

Diagnosis.

**Number and type of study designs reviewed:**

Thirteen method comparison studies and one prospective experimental study (fourteen total).

**Strength of evidence:**

Moderate.

**Outcomes reported:**

For oscillometry: two studies found it to be comparable; five studies found it to only be comparable in certain circumstances; and four studies found it not to be comparable (11 studies total measuring oscillometric). For Doppler: no studies found it to be completely comparable; two studies found it to be comparable under certain circumstances; and five found it not to be comparable (seven studies total measuring Doppler).

**Conclusion:**

The evidence is mixed and suggests that Doppler in cats is not comparable to the reference standard (invasive measurement) or may only be comparable under certain conditions. Oscillometry may be comparable to the reference standard but is also often found to only be comparable under specific conditions. There is more evidence for the comparability of oscillometry than for Doppler, particularly for measurement of mean arterial pressure.

How to apply this evidence in practice

The application of evidence into practice should take into account multiple factors, not limited to: individual clinical expertise, patient’s circumstances and owners’ values, country, location or clinic where you work, the individual case in front of you, the availability of therapies and resources.

Knowledge Summaries are a resource to help reinforce or inform decision making. They do not override the responsibility or judgement of the practitioner to do what is best for the animal in their care.

## Clinical Scenario

Assessing blood pressure is important in anaesthetised or critically ill cats to monitor for hypotension, and for older cats at risk of hypertension due to diseases such as chronic kidney disease or hyperthyroidism. Senior veterinary staff or practice managers may wish to know the accuracy of Doppler or oscillometric devices when making purchasing decisions.

In some clinical scenarios, the accuracy of a device may be less important than its internal consistency in order to monitor trends, such as response to medication. However, in some clinical scenarios a threshold of blood pressure is used to prompt a specific action even if there are no previous measurements for that patient, such as the criteria for beginning anti-hypertensive treatment in cats (Taylor et al., 2017) or treating hypotension under anaesthesia (Grubb et al., 2020). Therefore, it can still be important to know how closely the non-invasive blood pressure measurements match the invasive arterial method, which is the most accurate method available.

## The evidence

Thirteen method comparisons (diagnostic test evaluation studies) were analysed along with one prospective clinical study. Each compared a non-invasive method (Doppler or oscillometry) to the reference standard (direct arterial telemetry, also known as invasive). Three of these studies investigated only Doppler measurement (Cerejo et al., 2020, da Cunha et al., 2014; De Lombaert et al., 2023); seven investigated oscillometric measurement (Cerejo et al., 2017; Martel et al., 2013; Branson et al., 1997; Pedersen et al., 2002; Zwijnenberg et al., 2011; Acierno et al., 2010; Cremer et al., 2020); and four investigated both methods (Anjos et al., 2014; Binns et al., 1995; Caulkett et al., 1998; Haberman et al., 2006).

For Doppler measurement there was evidence suggesting that it is not comparable in cats, and some evidence suggesting it is only comparable under certain circumstances, particularly regarding the location and size of the cuff. For oscillometric there is some evidence that it is comparable, and other evidence that suggests it is only comparable for mean arterial pressure and not systolic pressure. Overall, the evidence is mixed and cannot substantiate that non-invasive blood pressure is accurate compared to the invasive method in cats, although there was more evidence for the comparability of oscillometric measurements than Doppler. This contrasts with the views of veterinarians; when surveyed, 69% of veterinarians believed Doppler to be more “trustworthy” than oscillometric measurements (Navarro et al., 2022). No change in clinical protocols would be suggested based on this evidence other than to take the cuff size and site of measurement into consideration when taking blood pressure measurements.

## Summary of the evidence

**Acierno et al. (2010):** Agreement between directly measured blood pressure and pressures obtained with three veterinary-specific oscillometric units in cats **Aim: **To compare oscillometric blood pressure to directly measured blood pressure.

Population:	Feline patients presenting for neutering to a Sterilisation Assistance Programme in the USA.
Sample size:	21 cats.
Intervention details:	Cats were sedated with midazolam (0.1 mg/kg) and ketamine (7 mg/kg) by intramuscular injection, and anaesthesia was induced and maintained with isoflurane. A 24G catheter was placed in a dorsal pedal artery and connected via a transducer to a monitor (Cardell Max-1). The pressure waveform was analysed before any readings taken. The dynamic response of the direct blood pressure monitoring system was tested in a canine patient before readings taken as the feline heart rate was too quick to test in the cat patients. Three oscillometric devices were used: 1) PetMap™ (Ramsey Medical), 2) VET HDO, (Vetline LLC) and 3) Cardell Max-1 (Sharn Veterinary Inc.). Devices 1 and 3 were used on the left antebrachium; device 2 was used on the tail. 4 paired measurements were taken 1 minute apart with each device. The direct reading was taken as the oscillometric cuff was deflating. The cuffs had a width of 30–35% of the limb circumference. No manipulation of blood pressure was performed but some results naturally fell in the hypotensive range.
Study design:	Method comparison study.
Outcome Studied:	Objective measurement of oscillometric blood pressure compared to directly measured.
Main Findings (relevant to PICO question):	For Device 1, the author did not consider it to be comparable but was not measuring against the American College of Veterinary Internal Medicine (ACVIM) values. Limits of agreement was displayed rather than standard deviation. The mean bias for mean arterial pressure (MAP) was -1.3 with a SD of < 15 mmHg, which would meet requirements. However, these were not met by systolic arterial pressure or diastolic arterial pressure. Devices 2 and 3 did not meet ACVIM requirements and were not considered comparable.
Limitations:	No power calculation was performed to inform suitability of sample size. Blood pressure was not manipulated since these were clinical patients, and results were not divided by blood pressure range. Device 3 only obtained values in 18/21 cats. Results were compared to an arbitrary value of 15 mmHg for mean bias and limits of agreement and this choice is not explained. Each device was only used in one location and they were not switched.

**Anjos et al. (2014):** Evaluation and comparison between methods of measurement of systolic blood pressure in healthy anesthetized cats **Aim: **To compare two methods of non-invasive systolic blood pressure (Doppler and oscillometric) to invasive blood pressure.

Population:	Healthy client-owned cats in Brazil.
Sample size:	12 cats.
Intervention details:	Cats were premedicated with acepromazine and meperidine, and anaesthetised with propofol, maintained with isoflurane. A right femoral arterial cannula was placed for invasive blood pressure (IBP) measurement, connected to a multiparameter machine. Using the PetMAP™ R monitor optimised for feline species, an oscillometric cuff was placed on the distal third of the right thoracic limb; using the manufacturer recommended size and seven measurements were taken 30 seconds apart and compared to IBP. The same cuff was then connected to a Doppler machine (Model 812(r), Parks Medical) and seven consecutive measurements were taken every 30 seconds compared to IBP taken at the same time. No artificial manipulation of blood pressure was performed.
Study design:	Method comparison study (experimental).
Outcome Studied:	Comparing two methods of non-invasive blood pressure (Doppler and oscillometric) to IBP (systolic arterial pressure only). Bland Altman method used for statistical analysis. Results assessed against Advancement of Medical Instrumentation (AAMI) standards.
Main Findings (relevant to PICO question):	Mean systolic arterial pressure (SAP) measured by Doppler was consistently lower than IBP. Doppler measurements were on average 12.58 mmHg lower than IBP (bias in this study was calculated as bias = reference method – test method). The Doppler results had high correlation (correlation coefficient 0.93), using linear regression analysis. The oscillometric measurements met AAMI standards for agreement with IBP regarding mean bias ≤ 5 mmgHg, which therefore also met American College of Veterinary Internal Medicine (ACVIM) standards. Standard deviation of the bias is not given.
Limitations:	Not tested across a wide range of blood pressure values and comparability can vary with hypotension or hypertension. Only one limb used (thoracic). Only one cuff size and cuff width to limb circumference ratio not provided. Cats were young, healthy and entire which is a skewed population compared to clinical patients, particularly with regard to diagnosis of hypertension. Did not discuss any additional ACVIM standards.

**Binns et al. (1995):** Doppler Ultrasonographic, Oscillometric Sphygmomanometric, and Photoplethysmographic Techniques for Noninvasive Blood Pressure Measurement in Anesthetized Cats **Aim: **To compare invasive blood pressure with three non-invasive methods in anaesthetised cats.

Population:	Adult mixed-breed cats at University of Illinois, Department of Veterinary Clinical Medicine, USA.
Sample size:	11 cats.
Intervention details:	Cats were anaesthetised using a combination of atropine, ketamine, diazepam, and isoflurane. A micro transducer (Miller Instrument Co.) for invasive blood pressure (IBP) was placed in the femoral artery or aorta. Non-invasive blood pressure using either Doppler, oscillometric or photoplethysmographic methods was recorded alongside a direct reading. The third method was not related to the PICO and will be excluded from this analysis. Doppler measurement was performed using a Parks Medical 811 device with a Critikon neonatal cuff; oscillometric was measured using the Dinamap Model 8300. Two operators performed the non-invasive measurements: one on the right hindlimb distal to the tarsus and one on the tail, using each non-invasive method in random sequence. Mean arterial pressure (MAP) was estimated where the Doppler method was used. A third person recorded the IBP. Blood pressure was artificially altered by changing anaesthetic depth, or with drugs (phenylephrine, dopamine or isoprenaline). Blood pressure ranges were defined as high (> 180 mmHg), medium (100–180 mmHg) and low (< 100 mmHg) using systolic readings. Five simultaneous measurements were taken, then this was repeated. The cuff width to limb circumference ratio was 0.35–0.45. If any machine failed to obtain a reading after 3 attempts or 10 minutes the attempt was abandoned. Linear regression analysis was used to calculate correlation coefficient. The difference (or bias) was calculated for each pair of measurements, as well as the percentage of measurements < 10 mmHg and 10–19.9 mmHg.
Study design:	Diagnostic test evaluation study.
Outcome Studied:	Comparison between invasive blood pressure and three non-invasive methods in anaesthetised cats.
Main Findings (relevant to PICO question):	No device met any American College of Veterinary Internal Medicine (ACVIM) criteria. Oscillometric method failed more often, particularly during hypotension. The highest bias (lowest agreement) was with oscillometric, and the lowest (best agreement) was with Doppler on the tail. Measurements were more comparable to the invasive method on the tail than on the distal hindlimb. Oscillometric measurements tended to underestimate compared to the invasive method (positive bias).
Limitations:	Data gathered only applies to anaesthetised cats. Information regarding the sex, bodyweight and exact age of the cats is missing, and their health status or source was not discussed. Therefore, there is limited analysis possible on their similarity to the general cat population. The name or type of oscillometric machine is not stated so it is unknown how likely the machine is going to be used in general veterinary practice. The authors found significant respiratory fluctuations in blood pressure during light anaesthesia (up to 20 mmHg), which may have affected reliability of measurements. There was no power calculation.

**Branson et al. (1997):** Evaluation of an oscillometric blood pressure monitor on anesthetized cats and the effect of cuff placement and fur on accuracy **Aim: **To determine the accuracy of a specific oscillometric machine compared to invasive blood pressure, and the effect of measurement site and fur clipping on the accuracy.

Population:	Adult mixed-breed shorthair cats at the University of Missouri, USA.
Sample size:	6 cats.
Intervention details:	The cats were premedicated with acepromazine and anaesthetised with isoflurane. The carotid artery was cannulated and connected to a CDX transducer (Cobe Cardiovascular Inc.) which was placed at the level of the heart. Oscillometric measurements of blood pressure were taken with the Datascope Passport machine on neonatal mode at two sites: the median artery distal to the elbow and the cranial tibial artery distal to the stifle. The hair at the measurement site on the right limbs was clipped, and the left sides were left unclipped. A cuff with 40–60% of limb circumference was used. Blood pressure (BP) was manipulated by increasing anaesthetic depth or giving a dobutamine infusion to achieve the following ranges of mean arterial pressure (MAP): 80–100 mmHg (normotension), 40–60 mmHg (hypotension), and 120–140 mmHg (hypertension). Approximately 35 simultaneous measurements of invasive blood pressure (IBP) and oscillometric BP were taken from each blood pressure range on each forelimb and hindlimb, including systolic, diastolic, and mean pressure.
Study design:	Method comparison study (experimental).
Outcome Studied:	The accuracy of a specific oscillometric machine compared to IBP, and the effect of measurement site and fur clipping on the accuracy of the measurement.
Main Findings(relevant to PICO question):	The mean bias of the two methods ranged from 8.1–34.4 mmHg depending on location of the cuff and the BP itself. The oscillometric measurements were not considered comparable or correlated and consistently underestimated the IBP. Oscillometric measurements failed more often during hypotension.
Limitations:	Sample size was small and did not include a power calculation. The sex of the cats was not stated. These were laboratory cats which may differ from the companion cat population. The results only apply to anaesthetised cats. The oscillometric machine was a human one adapted for veterinary use and may not reflect what is used in general practice. The ratio of cuff size to limb circumference was most different to other studies and outside the current recommendation, which could have affected measurements.

**Caulkett et al. (1998):** A Comparison of Indirect Blood Pressure Monitoring Techniques in the Anesthetized Cat **Aim: **To measure agreement between three methods of non-invasive blood pressure measurement and the invasive method in cats.

Population:	Adult cats at University of Saskatchewan, Canada.
Sample size:	8 cats.
Intervention details:	Cats were anaesthetised with isoflurane. A pressure transducer was connected to a cannula placed into the left femoral artery and used to measure Invasive blood pressure (IBP). Doppler BP (Model 811-AL; Parks Medical Inc.) was measured on the forelimb between the carpus and the elbow, and oscillometric (Veterinary Dinamap machine) was measured in the same location on the opposite forelimb. A third method was measured not relevant to the PICO which will not be discussed. Isoflurane concentration was adjusted to achieve a direct systolic BP of 80–100 mmHg (mild hypotension) for 30 minutes and recordings were taken every 5 minutes. The same procedure was repeated for 60–80 mmHg (moderate hypotension) and < 60 mmHg for only 15 minutes (severe hypotension). Two operators measured the indirect blood pressure, who were blinded to the IBP. The data was analysed using the Bland Altman technique.
Study design:	Method comparison study (experimental).
Outcome Studied:	Objective measurement of agreement between three methods of non-invasive blood pressure measurement and the invasive method.
Main Findings (relevant to PICO question):	No non-invasive methods met the American College of Veterinary Internal Medicine (AVCIM) standards; mean bias was –15.9 mmHG for oscillometric measurements mmHg and –25 mmHg for Doppler measurements. All methods underestimated systolic pressure compared to the IBP. However, when comparing Doppler systolic to invasive mean arterial pressure, there was no statistical difference and mean bias was –0.8 mmHg, which would meet ACVIM standards. When hypotension was severe, oscillometric measurements of systolic arterial pressure (SAP) failed in 21 of 40 attempts, and mean arterial pressure (MAP) failed in 8 of 40 attempts. This method failed most often.
Limitations:	The methods describe the femoral artery being cannulated, but the discussion mentions the IBP being in a ‘central’ location i.e. the aorta, which is a discrepancy. The age or health status of the cats is not stated. BP was not manipulated to produce hypertension, only hypotension. Data is only applicable to anaesthetised cats. There is no power calculation to inform sample size suitability. Only one location (the forelimb) was used for non-invasive measurement. To reduce the risk of adverse effects, the number of measurements during severe hypotension was lower.

**Cerejo et al. (2017):** Comparison of two species-specific oscillometric blood pressure monitors with direct blood pressure measurement in anesthetized cats **Aim: **To compare measurement of blood pressure via two different oscillometric machines to invasive blood pressure.

Population:	Client-owned adult cats presented for neutering at a veterinary teaching hospital in the USA.
Sample size:	8 cats.
Intervention details:	Cats were premedicated with ketamine, midazolam, and methadone, and then anaesthetised with propofol and maintained with isoflurane gas. Invasive blood pressure (IBP) was performed by telemetry via a dorsal pedal artery cannula connected to a calibrated pressure column. Two different PetMAP™ oscillometric machines used (Classic and Graphic); one cuff placed on a thoracic limb and one on the tail. Cuff size to limb circumference ratio was 0.45 ± 0.02 in the thoracic limb and 0.44 ± 0.04 in the tail, with cuffs selected according to manufacturer instructions. IBP was adjusted pharmaceutically to produce a range of IBP values in approximately 10 mmHg increments; hypotension was induced by increasing isoflurane and hypertension was induced by decreasing isoflurane and an intravenous dopamine infusion. Norepinephrine was used in one cat and phenylephrine was used in one cat. At each blood pressure level three measurements with each oscillometric machine were taken simultaneously to IBP when IBP was stable for 5 minutes (not more than 5 mmHg variation). Three measurements were then taken with the cuffs at the opposite location (tail and limb switched). Any failures of the oscillometric machine to achieve a reading were recorded.
Study design:	Method comparison study (experimental).
Outcome Studied:	Objective measurement of blood pressure via two different oscillometric machines compared to IBP. Mean bias (test method – reference method) and limits of agreement (LOA = 1.96 x SD) calculated according to the modified Bland Altman method. Results compared to standards set by the Association for the Advancement of Medical Instrumentation (AAMI) (mean bias ≤ 5 mmgHG and SD ≤ 8 mmHG) and the American College of Veterinary Internal Medicine (ACVIM) standards (mean bias ≤ 10 mmHg and SD ≤ 15 mmHg).
Main Findings (relevant to PICO question):	Agreement of mean arterial pressure (MAP) measured by both oscillometric machines was acceptable compared to invasive measurement by AAMI standards (mean bias ≤ 5 mmHg with LOA ≤ ± 16 mmHg). Agreement of systolic arterial pressure (SAP) between direct and oscillometric methods did not meet AAMI or ACVIM standards; the oscillometric method tended to overestimate systolic pressure.
Limitations:	Results can only be applied to cats under anaesthesia. Maximum MAP of 105 mmHg achieved with drug intervention which may not reflect a true hypertensive state. Conclusions limited to thoracic limb and tail only. No power calculation for discussion of sample size. Pharmaceutical intervention for inducing hypertension not the same in every cat; standardised approach would have been preferable as no specific explanation given for the differences. Machine Classic (C) failed to provide a reading on 5 occasions; machine Graphic (G) failed to provide a reading on 27 occasions. Both were associated with hypotension. This could result in a survivorship type bias where results could have been different if those measurements succeeded.

**Cerejo et al. (2020):** Effects of cuff size and position on the agreement between arterial blood pressure measured by Doppler ultrasound and through a dorsal pedal artery catheter in anesthetized cats **Aim: **To determine the effect of cuff size and cuff location on agreement between Doppler measurement and invasive blood pressure.

Population:	Client-owned cats presented for neutering at veterinary teaching hospital in Brazil.
Sample size:	8 cats.
Intervention details:	Cats were premedicated with midazolam, ketamine and morphine, and anaesthetised with propofol, maintained by isoflurane gas. Dorsal pedal artery cannulation performed for measurement of invasive blood pressure (IBP). The Parks Medical Inc. Doppler device (Model 811-B) was used for non-invasive measurements. Three different cuff sizes used, with cuff width to limb circumference ratio measured. Doppler measurements performed with the cuff at three locations: distal forelimb and hindlimb (above and below tarsus). Blood pressure was manipulated by adjusting isoflurane concentration and administering dopamine to achieve 9 ranges of invasive systolic arterial pressure (SAP) between 60 mmHg and 150 mmHg. Three serial oscillometric measurements for each cuff size were performed alongside IBP in each blood pressure range; one person performing invasive and one person performing Doppler. Surgical neutering was performed after the end of the experiment.
Study design:	Method comparison study (experimental).
Outcome Studied:	Effect of cuff size and location on agreement between Doppler measurement and invasive blood pressure.
Main Findings(relevant to PICO question):	Advancement of Medical Instrumentation (AAMI) criteria for agreement between Doppler and IBP (mean bias ≤ 5 mmgHg and SD ≤ 8 mmHg) were not met by any cuff size or position. SAP by Doppler met American College of Veterinary Internal Medicine (ACVIM) standards (mean bias ≤ 10 mmHg and SD ≤ 15 mmHg) for agreement with invasive methods with cuff sizes 1 and 2 on the thoracic limb and cuff size 2 placed above the tarsus. SAP measured by Doppler did not provide a good estimation of mean arterial pressure (MAP) (measured by IBP) as proposed by some authors. SAP measurements did not meet ACVIM standards for any measurements taken distal to the tarsus with any cuff size. SAP measurements taken with cuff size 3 did not meet ACVIM standards in any location.
Limitations:	Cats were young, healthy and entire, which is a narrow population subset. The full 9 ranges of systolic blood pressure ranges were only achieved in 4 of the 8 cats; in 2 cats there were 8 ranges achieved and in 2 cats only 7. Results can only be applied to anaesthetised cats. Systolic blood pressure range achieved by drug intervention did not reach the range of 150–200 mmHg, and therefore was not high enough to reflect a conscious hypertensive cat. No power calculation to inform sample size suitability. Non-invasive measurement was not performed on the tail which is common in the clinical setting. The minimum natural frequency required for accurate invasive blood pressure waveform display was not achieved in 1 of 8 cats which could have affected the comparability of the IBP.

**Cremer et al. (2020):** Validation of the oscillometric blood pressure monitor Vet20 Suntech in anesthetized healthy cats **Aim: **To compare oscillometric blood pressure to invasive measurements, and to attempt to validate a specific oscillometric device (Vet20 machine, Suntech Medical).

Population:	Shelter cats presenting for neutering at Louisiana State University, USA.
Sample size:	33 cats.
Intervention details:	Anaesthesia was induced with isoflurane after premedication with methadone and alfaxalone. A 22 gauge, 2.5 cm catheter (Surflo I.V. Catheter; Terumo Medical Corp.) was inserted into the medial caudal artery. Oscillometric measurements were taken on the antebrachium with the Vet20 machine (SunTech Medical Inc.) A cuff with 40% width of the limb circumference was used. Five paired recordings of systolic, diastolic and mean pressure were taken at 5-minute intervals and an average taken of these. Any failure of the non-invasive device or repositioning of the cuff was recorded. No artificial manipulation of blood pressure was performed, except that hypotension (mean arterial pressure (MAP) < 60 mmHg) was treated at the discretion of an anaesthesiologist. Neutering was performed after the recordings. Bland Altman analysis and Pearson correlation coefficient were performed on the data. 3 cats were excluded from the initial 33 as it was not possible to place an arterial catheter.
Study design:	Method comparison study.
Outcome Studied:	Objective measurement of oscillometric blood pressure compared to invasive measurements, to attempt to validate a specific machine.
Main Findings (relevant to PICO question):	5 of the 6 American College of Veterinary Internal Medicine (ACVIM) criteria were met by this machine; only the correlation did not meet the standards (was < 0.9 in this study). Mean bias and standard deviation were acceptable for systolic arterial pressure, diastolic arterial pressure, and MAP. The oscillometric device did not fail to obtain a reading at any time. The power of the study was 90% using a standard technique and showed the sample size was suitable to make the conclusions stated.
Limitations:	Only the forelimb was used to measure non-invasive blood pressure, so results only apply to this one location. Results only apply to anaesthetised cats since measurements were only performed under general anaesthetic. Blood pressure was not manipulated so only a minority of readings fell naturally into the hypertensive range (defined as SAP > 125 mmHg). Therefore, there can be limited conclusions drawn about the agreement during hypertension.

**da Cunha et al. (2014):** Measuring level of agreement between values obtained by directly measured blood pressure and ultrasonic Doppler flow detector in cats **Aim: **To measure the agreement between Doppler blood pressure measurement and two sites of invasive blood pressure measurement.

Population:	Adult shelter cats presented for elective sterilisation at Louisiana State University Animal Sterilization Assistance Program.
Sample size:	39 cats.
Intervention details:	Cats were anaesthetised with midazolam, ketamine, hydromorphone and then isoflurane. Cats were then placed into two groups: A and B. Group A had a dorsal pedal artery catheter placed connected to a pressure transducer for invasive measurement. Group B had a femoral catheter placed with a different diameter catheter and different type of transducer for invasive blood pressure (IBP). Both groups had Doppler measurements taken with a Parks Medical (Model 811-B) device, on the distal third of the forelimb with a cuff width 30–40% of the limb circumference. One person obtained indirect Doppler measurements who was blinded to the invasive result taken at the same time. Results were split based on the mean arterial pressure (MAP): hypotensive (MAP < 60 mmHg), normotensive (60–90 mmHg), hypertensive (> 90 mmHg) but not manipulated artificially. Measurements were repeated 4 times at 1-minute intervals.
Study design:	Diagnostic test evaluation study.
Outcome Studied:	Objective measurement of agreement between Doppler blood pressure measurement and 2 sites of invasive blood pressure measurement.
Main Findings (relevant to PICO question):	One cat was excluded due to having outlier results. Mean bias for systolic, mean, and diastolic arterial pressures were -8.8 mmHg, 14 mmHg, and 27.9 mmHg respectively. Doppler measurements compared poorly to the invasive reference standard and also had poor correlation. They did not meet Advancement of Medical Instrumentation (AAMI) or American College of Veterinary Internal Medicine (ACVIM)standards. Methodology, bodyweight, or sex did not affect the agreement.
Limitations:	One location used for measurement only (forelimb). Randomisation of assignment to groups not discussed, and if not performed could introduce selection bias which could affect results. Doppler machine was a paediatric unit and not veterinary specific. Study does not state whether the experiment was performed during or before the neutering surgery. Does not discuss whether the person performing measurements was experienced in Doppler blood pressure.

**De Lombaert et al. (2023):** Effect of gabapentin on ambulatory, direct, systemic arterial blood pressure in apparently healthy cats in the at-home and in-clinic environments **Aim: **To measure the effect of gabapentin on direct arterial blood pressure in cats at home and in a veterinary clinical environment.

Population:	Purpose-bred male neutered domestic shorthair cats between 2 and 3 years of age at University of Georgia, USA.
Sample size:	5 cats (one excluded after an initial 6).
Intervention details:	Laboratory cats were acclimatised to their environment for 30 days then an arterial telemetry device was surgically implanted into the femoral artery (anaesthesia protocol not described). Cats recovered for 21 days before data collection. Initial study phase involved invasive blood pressure (IBP) only and was not relevant to the PICO, the second phase involved a simulated veterinary clinic visit which was relevant to the PICO. In phase two, cats were given either 100 mg of gabapentin or a placebo orally and transported to a veterinary clinic 90 minutes later by car. Cats waited in a waiting room for 5 minutes and had IBP measured throughout. In an exam room, Doppler systolic blood pressure measurements were taken on the tail over a 10-minute period, while simultaneous IBP recordings were taken. The first Doppler measurement was discarded leaving 5 valid measurements. The Model 811-B; Parks Medical device was used for Doppler measurements.
Study design:	Prospective crossover experimental study.
Outcome Studied:	Effect of gabapentin on direct arterial blood pressure in cats at home and in a veterinary clinical environment.
Main Findings (relevant to PICO question):	The mean Doppler SBP was 15.6 mmHg lower than the SAP (equivalent to a mean bias), which does not meet American College of Veterinary Internal Medicine (ACVIM) standards (mean bias ≤ 10 mmHg). The Doppler measurement did not agree with mean arterial pressure (MAP) either.
Limitations:	Comparison of IBP to Doppler was not the main aim of the study so statistical analysis is not specific to this aim; the Bland Altman method was not used. The telemetry arterial device failed in one cat of six leaving only five for analysis. Sample size was small and a power calculation was not performed. This was a skewed sample of young, male, castrated cats kept under laboratory conditions, and results apply only to that population which does not represent the overall companion cat population. There was no artificial alteration of blood pressure to achieve hypo- or hypertensive states so the accuracy of the non-invasive method in those states cannot be determined. Only one location (the tail) was used for Doppler measurement.

**Haberman et al. (2006):** Evaluation of Doppler ultrasonic and oscillometric methods of indirect blood pressure measurement in cats **Aim: **To measure Doppler and oscillometric blood pressure compared to invasive blood pressure in conscious and anaesthetised cats.

Population:	Adult mixed breed laboratory cats that were health tested, at University of Georgia (USA).
Sample size:	13 cats.
Intervention details:	A pilot study was performed to determine the site of strongest correlation of both methods with invasive blood pressure, which involved the median and tibial arteries with Doppler, and the median, tarsal and coccygeal arteries with oscillometric. For oscillometric measurements, median and coccygeal arteries had the best correlation; for Doppler measurements the median artery was best. Cats were anaesthetised with thiopental and maintained with halothane; the femoral artery was catheterised, and a pressure transducer that transmitted a radio signal was placed in the ventral subcutaneous fat. Some cats also had a partial nephrectomy to induce hypertension. Non-invasive blood pressure (NIBP) measurements were taken using a neonatal cuff size 2 which was 30–50% of limb circumference. Oscillometric measurements were recorded with a Dinamap Model 8300 machine (Critikon) at the two sites: forelimb and tail. Doppler measurements were taken with a Model 811 Parks Electronics machine on the forelimb. Comparative studies were performed conscious on the 13 cats; and performed under anaesthesia in 4 of the cats. It was unclear if these 4 cats were part of the same group as the 13 or a different group. For conscious studies, cats were held on a table for 5 minutes where the receiver was located before measurements began. Cats that had nephrectomy performed did not receive any drugs; normal cats received either atenolol or hydralazine twice daily to produce a drop of 20 mmHg in mean arterial pressure (MAP). A minimum of 5 measurements of each NIBP method were taken before and after the drugs were given; any taken while the cat was moving were discarded. For measurements under anaesthesia, blood pressure (BP) was lowered by increasing inspired halothane concentration and taking 3–5 paired measurements with each method at each 10mmHg reduction, until MAP was 50 mmHg. Blood pressure was then increased by infusing lactated Ringer’s solution with phenylephrine intravenously and taking paired measurements with each method every 10 mmHg increase until it was 30 mmHg above baseline.
Study design:	Method comparison study.
Outcome Studied:	Objective measurement of Doppler and oscillometric blood pressure compared to invasive blood pressure in conscious and anaesthetised cats.
Main Findings (relevant to PICO question):	For conscious cats, neither method met any American College of Veterinary Internal Medicine (ACVIM) standards, although using the average of 5 measurements increased the correlation. For anaesthetised cats, neither method met ACVIM standards but Doppler was closer to meeting them with a mean bias of 11.5 mmHg. Results for oscillometric and Doppler measurements were more comparable under anaesthesia than conscious. Doppler BP was more correlated with IBP than oscillometric BP in conscious and anaesthetised cats. The oscillometric method failed to obtain a measurement more often than the Doppler (the failure rate for Doppler was zero), which varied between individual cats but not between the 2 sites.
Limitations:	A power calculation was not performed. The nephrectomy method of inducing hypertension is invasive and may not model hypertension more reliably than giving a drug. The conscious studies were performed with 13 cats; the measurements under anaesthesia were performed in 4 cats but it is not clear whether these are 4 separate cats or part of the same group of 13. Cuff size to limb circumference ratio varied widely which may have reflected results. Randomisation of cats to receive nephrectomy or not was not discussed. Not stated whether the person performing the measurements was blinded to the other method, or how many people performed it. During adjustments of BP under anaesthesia, the induction of hypertension was described in increments above baseline but the actual values were not given.

**Martel et al. (2013):** Comparison of high-definition oscillometry — a non-invasive technology for arterial blood pressure measurement — with a direct invasive method using radio-telemetry in awake healthy cats **Aim: **To compare high definition oscillometry to invasive telemetry in conscious cats.

Population:	Purpose-bred experimental juvenile cats in Europe.
Sample size:	6 cats.
Intervention details:	Pressure sensor cannula implanted in abdominal aorta via the femoral artery under general anaesthesia (using buprenorphine and propofol), with a receiver placed subcutaneously. Two week waiting period to acclimatise to group housing conditions. High Definition Oscillometric (HDO;S + B MedVET) device used, with the cuff on the tail. Five sets of 5 simultaneous oscillometric invasive blood pressure (IBP) readings per day per cat. Amlodipine or phenylephrine given orally to achieve a set range of blood pressures.
Study design:	Method comparison study (experimental).
Outcome Studied:	Comparison between objective measurements of blood pressure by high definition oscillometry and invasive telemetry in conscious cats.
Main Findings (relevant to PICO question):	The bias of oscillometric systolic arterial pressure (SAP)compared to invasive met the American College of Veterinary Internal Medicine (ACVIM) standard when SAP was normal or high, but was just outside the standards when blood pressure was low. Additional ACVIM criteria were met: 50% of measurements with a difference of ≤ 10 mmHg or 80% of measurements with a difference of ≤ 20 mmHg, but difference was wider as blood pressure increased. Part of the Advancement of Medical Instrumentation (AAMI) standards (mean bias ≤ 5 mmgHg and standard deviation (SD) ≤ 8 mmHg) were met by high definition oscillometry. Additional AAMI standards (95% of measurements to be within 10mmHg of the reference method) were not met. Diastolic arterial pressure did not meet ACVIM standards for agreement.
Limitations:	Small sample size and no power calculation, fewer than 8 which is part of the ACVIM guidelines for validation of an indirect method. Population was purpose bred, young, and healthy which may not accurately reflect the type of cats that are at risk of hypertension. Cats were well-handled, group housed and acclimatised to the measurements which may not apply well to clinical patients who can experience more stress from the measurement of blood pressure. Therefore the ‘white coat’ effect may be minimised in this study. Results can only be applied to conscious cats using the coccygeal artery on the tail. High definition oscillometry may not be widely available in veterinary practice and results only apply to this machine.

**Pedersen et al. (2002):** Evaluation of an oscillometric blood pressure monitor for use in anesthetized cats **Aim: **To measure the accuracy of an oscillometric device in anaesthetised cats.

Population:	Persian cats with subclinical polycystic kidney disease at The Royal Veterinary and Agricultural University, Frederiksberg, Denmark.
Sample size:	6 cats.
Intervention details:	The cats were anaesthetised with a combination of tiletamine, zolazepam, xylazine, and butorphanol, then maintained with isoflurane. The right femoral artery was cannulated with a 22-gauge 3.49 cm polyurethane arterial catheter (Teleflex, Arrow) and connected to a transducer (Px260, Baxter). Oscillometric measurements were taken on the left forelimb at antebrachium after clipping the fur with a neonatal cuff displayed on a Cardell 9301V monitor. The mean ratio of cuff width to limb circumference was 0.37. Simultaneous measurements of oscillometric and invasive blood pressure were taken every 2 minutes 10 consecutive times during normotension. Blood pressure was manipulated by adjusting isoflurane or administering an alpha 2 agonist or dopamine infusion. Simultaneous measurements were taken 5 times during hypotension (mean arterial pressure (MAP) < 60 mmHg) and hypertension (MAP > 140 mmHg). The cats were not recovered from the procedure.
Study design:	Method comparison study.
Outcome Studied:	An objective measurement of the accuracy of an oscillometric device in anaesthetised cats.
Main Findings (relevant to PICO question):	Mean bias between the methods was < 5 mmHg for diastolic arterial pressure (DAP) and MAP in all pressure ranges. Standard deviation was < 8 mmHg in all measurements. Therefore, DAP and MAP measurements met (ACVIM) and the more stringent Advancement of Medical Instrumentation (AAMI) criteria for accuracy. Both standards were also met by measurements of systolic arterial pressure (SAP) during hypotension only. Measurements of SAP during normotension and hypertension did not meet ACVIM standards. As SAP increased, it was increasingly underestimated.
Limitations:	The sample size was only 6 and no power calculation was performed. The population was only of one breed and (although clinically healthy) had ultrasonic evidence of kidney disease which makes this a skewed sample of the cat population. Oscillometric measurements were only taken at one site. Data only applies to anaesthetised cats.

**Zwijnenberg et al. (2011):** Evaluation of oscillometric and vascular access port arterial blood pressure measurement techniques versus implanted telemetry in anesthetized cats **Aim: **To compare blood pressure measured by non-invasive oscillometry and a semi-invasive vascular access port with invasive telemetry in cats.

Population:	Healthy domestic shorthair cats aged from 12 to 17 months in Australia.
Sample size:	6 cats.
Intervention details:	Under isoflurane anaesthesia, an arterial catheter was placed in the abdominal aorta via the left femoral artery (day 0). The associated radiotransmitter was secured in the flank abdominal muscle. Baseline measurements of invasive blood pressure (IBP) were taken when the cats were awake, but not with a non-invasive method. The cats were anaesthetised for measurements at week 4 and 14, as well as week 12 and 13 where minimum alveolar concentration (MAC) of isoflurane was being determined. Oscillometric measurements were taken on the tail; IBP measurements were taken immediately prior to cuff inflation. The Cardell veterinary monitor, model 9403 was used; cuff sizes 2–2.5cm were used. Paired readings were taken at normotension (mean arterial pressure (MAP) < 85 mmHg) after a stabilisation period of 15 minutes. Hypertension (> 85 mmHg) was induced by administering a phenylephrine bolus and paired measurements were also taken then.
Study design:	Method comparison study.
Outcome Studied:	Objective comparison of blood pressure measured by non-invasive oscillometry and a semi-invasive vascular access port with invasive telemetry in cats.
Main Findings (relevant to PICO question):	The mean bias for MAP measured by oscillometric was 10.0 ±3 mmHg and correlation was high at 0.917 which would meet American College of Veterinary Internal Medicine (ACVIM)standards, although not the more stringent Advancement of Medical Instrumentation (AAMI) standards. Systolic and diastolic pressure accuracy did not meet ACVIM standards but correlation was high, and close to meeting standards (0.896 and 0.898 respectively). Bias for systolic arterial pressure (SAP) did not meet but was close to meeting the ACVIM standards at -10.1 ± 16.7 mmHg.
Limitations:	There was no power calculation and the sample size is lower than necessary for device validation by the ACVIM. The cuff size to limb circumference ratio is not stated which is known from other studies to affect the accuracy. Data only applies to anaesthetised cats as no non-invasive measurements were taken while cats were conscious. Oscillometric measurements were only taken at one site (the tail). Recordings were not taken when the cats experienced hypotension. Data does not compare the results between the normotensive and hypertensive states.

## Appraisal, application and reflection

This Knowledge Summary evaluates two test methods of measuring blood pressure (oscillometric and Doppler) against the method that is considered the most accurate method available (invasive arterial pressure), even if no method is truly 100% accurate. For this reason, the word comparable is mostly used in the summary. Where the term accuracy is used (for example, because the study or author being referred to also used the term) it refers to the accuracy of the device compared to the reference method (invasive method).

No systematic review articles were found in the literature search for this topic, which would be the highest level of evidence. All the articles in this Knowledge Summary except one are Level 1B (a validating cohort study) which is the next best evidence level for a study investigating diagnosis (Howick et al., 2009) The remaining study (De Lombaert et al., 2023) is an exploratory cohort study, which is Level 2 evidence equivalent to a randomised controlled trial (Howick et al., 2009).

The American College of Veterinary Internal Medicine (ACVIM) published guidelines for validation of a non-invasive blood pressure device in animals (Brown et al., 2007), which are based on the American Association of Medical Instrumentation (AAMI) criteria but less stringent and more appropriate for devices in veterinary patients. The only more recent guidance published by the AAMI in conjunction with other relevant organisations is for human devices exclusively (Stergiou et al., 2018), therefore the ACVIM standards were the most appropriate. The main standard is that the mean bias between the methods should be ≤ 10 mmHg and standard deviation (SD) ≤ 15 mmHg, which is the basis of accuracy assessment in this summary. None of the papers in this summary found the non-invasive device met all the standards for validation of the method, but some met this main standard. One paper (Cremer et al., 2020) found that the non-invasive oscillometric device met 5 of 6 standards set by ACVIM, only failing for correlation which was considered to be of least importance.

Of the seven papers assessing the Doppler method (Cerejo et al., 2020; Anjos et al., 2014; da Cunha et al., 2014^, ^; De Lombaert et al., 2023; Binns et al., 1995; Caulkett et al., 1998; Haberman et al., 2006), five found it to be not comparable (Anjos et al., 2014 ; da Cunha et al., 2014; De Lombaert et al., 2023; Binns et al., 1995; Haberman et al., 2006) and two found it to be comparable only under certain circumstances (Cerejo et al., 2020, and Caulkett et al., 1998). For example, Cerejo et al. (2020) found that systolic arterial pressure (SAP) by Doppler measurement met ACVIM standards (mean bias ≤ 10 mmHg and SD ≤ 15 mmHg) for agreement with invasive methods only with cuff sizes 1 and 2 on the thoracic limb and cuff size 2 placed above the tarsus. It has been suggested that in cats, measurement of SAP by Doppler may actually be a closer estimate of mean arterial pressure (MAP) because it underestimates SAP (Caulkett et al., 1998). However, this summary did not substantiate that finding as two authors found this to be untrue (De Lombaert et al. 2023; Cerejo et al., 2020).

Plethysmography was not reviewed in this summary as it is currently an uncommonly used non-invasive method in general veterinary practice (Skelding & Valverde, 2020).

Of the eleven papers assessing the oscillometric method (Cerejo et al., 2017; Anjos et al., 2014; Martel et al., 2013;Binns et al., 1995;Caulkett et al., 1998; Branson et al., 1997; Pedersen et al., 2002; Haberman et al., 2006; Zwijnenberg et al., 2011; Acierno et al., 2010; Cremer et al., 2020) two found it to be comparable (Anjos et al., 2014 and Cremer et al., 2020) and four found it to be not comparable (Binns et al., 1995; Caulkett et al., 1998; Branson et al., 1997; Haberman et al. 2006). However, five found it to be comparable only under certain circumstances (Cerejo et al., 2017; Martel et al., 2013; Pedersen et al., 2002; Zwijnenberg et al., 2011; Acierno et al., 2010). In these circumstances, Cerejo et al. (2017) found oscillometry only to be accurate for MAP and not SAP. Martel et al. (2013) found oscillometry to be not comparable during hypotension and for diastolic pressure. In contrast, Pedersen et al. (2002) found that SAP measured by oscillometry was comparable only during hypotension, while MAP was consistently comparable. Zwijnenberg et al. (2011) found that oscillometric measurements were not comparable for systolic or diastolic pressure, and only comparable for MAP.

Of the four papers that evaluated the Doppler and oscillometric method (Anjos et al., 2014; Binns et al., 1995; Caulkett et al., 1998; Haberman et al. 2006), one (Anjos et al., 2014) found that although Doppler measurements were not comparable, they were highly correlated, such that a correction factor could be applied to the results allowing Doppler to be used in a manner that would be more similar to the invasive method. These findings were not replicated by da Cunha et al. (2014) who found Doppler measurements to be poorly correlated.

There are several limitations to the studies discussed in this summary. Firstly, the cat populations and their source was varied. They consisted of laboratory cats (Martel et al., 2013; de Lombaert et al., 2023 Binns et al., 1995; Branson et al., 1997; Haberman et al., 2006; Zwijnenberg et al., 2011) which would be considered convenience sampling, shelter cats (da Cunha et al., 2014; Cremer et al., 2020), pet cats presented for neutering (Cerejo et al., 2017; Cerejo et al. 2020; Acierno et al., 2010) which is a type of consecutive sampling, tutor-owned cats (Anjos et al., 2014), ex breeding cats presented for euthanasia (Pedersen et al., 2002), and adult cats with no further specification (Caulkett et al., 1998). Most were young and considered healthy by physical exam, with or without health screening, creating a skewed sample that does not fully represent the pet cat population.

Secondly, several different arteries were used to measure both invasive and non-invasive blood pressure used across the papers. The study that investigated this specifically (Cerejo et al., 2020) found that the location of the non-invasive measurement affected the comparability of the readings significantly; any measurements taken in the hindlimb below the tarsus did not meet ACVIM standards for accuracy. The locations used for non-invasive measurement included the forelimb only in 5 papers (Anjos et al., 2014; da Cunha et al., 2014; Caulkett et al., 1998; Pedersen et al., 2002; Cremer et al., 2020), tail only in 3 papers (Martel et al., 2013; De Lombaert et al., 2023; Zwijnenberg et al., 2011), the forelimb and tail in 3 papers (Cerejo et al., 2017; Haberman et al., 2006; Acierno et al., 2010) and another combination in the remaining 3 papers (Cerejo et al. 2020; Binns et al., 1995; Branson et al., 1997).

The dorsal pedal artery, femoral artery, and abdominal aorta were all used for invasive measurement, which could achieve different results for the reference standard; this limitation was discussed by Martel et al. (2013). It has been shown in dogs (Monteiro et al., 2013) and horses (Midon et al., 2023) that results for invasive pressure from different arteries vary. However, one of the papers in this Knowledge Summary investigated both femoral and dorsal pedal arteries in different groups for invasive measurement and found no significant difference between them (da Cunha et al., 2014), so it is possible that smaller size or specific species differences mean this is not relevant to cats.

There was a great variety of equipment used in the papers analysed, including nine different multiparameter monitors for oscillometric measurement and several different cannulas and transducers. This introduces variability that is not controlled for. All seven papers evaluating the Doppler method (Anjos et al., 2014; da Cunha et al., 2014; De Lombaert et al., 2023, Binns et al., 1995; Caulkett et al., 1998; Cerejo et al., 2020; Haberman et al., 2006) used a Parks Medical Device, of which six were exactly the same model. However, only one paper of these seven was able to establish any agreement with the invasive method. All three studies that used a Dinamap device (Binns et al., 1995; Caulkett et al., 1998; Haberman et al., 2006) found oscillometry not to be comparable to invasive. In contrast, the three times a petMAP™ device was used (different specific models), it was found to agree with the invasive method for MAP every time, even if not for SAP and DAP. A Cardell device featured three times in different papers (with different specific models) and was found to be comparable for MAP in two of the three studies (Zwijnenberg et al., 2011; Pedersen et al., 2002). Other devices were only tested once across all the papers and varied in whether they could be validated as accurate compared to the invasive method. Some newer popular multiparameter devices using oscillometry such as the Mindray or Lutech Datalys did not feature in any of the studies in this summary.

Agreement between invasive measurements in different arteries can vary depending on the haemodynamic situation of the animal, such as the heart rate (Monteiro et al., 2013). This could be profoundly affected by choice of anaesthetic drugs, and the varied regimes in these papers could account for some differences in the accuracy.

Thirdly, the accuracy of non-invasive devices can vary with the blood pressure value itself (Garofalo et al., 2012), so it is important to measure the agreement with a reference standard across a range of values. Multiple papers (Anjos et al., 2014; da Cunha et al., 2014; De Lombaert et al., 2023; Acierno et al., 2010; Cremer et al., 2020) did not attempt to manipulate blood pressure to achieve a range of measures. These papers cited ethical reasons for not performing this as hypotension can cause harm and the cats were pets. Papers in this summary that did manipulate blood pressure artificially either obtained informed consent and kept the hypotensive states as short as possible or performed the experiment under terminal anaesthesia. Ethical views on this may vary between countries and institutions. There was little consistency of the method used to manipulate blood pressure, and of the ranges used to define hypo-, hyper- and normotension.

Furthermore, diagnosis of hypertension in conscious cats is an important application of BP measurement and only three papers (Martel et al., 2013; De Lombaert et al., 2023; Haberman et al., 2006) measured BP in conscious animals, only one of which was with Doppler measurements (De Lombaert et al., 2023). Since a device can only be validated for the conditions under which it was studied (Brown et al., 2007), data from eleven studies can only be applied to anaesthetised cats which is a significant limitation. Under anaesthesia it is difficult to achieve blood pressures in the hypertensive range that would mimic a clinical patient, even with pharmaceutical manipulation. Two of the papers in this summary specifically mentioned difficulty with this (Cerejo et al., 2017; Cerejo et al., 2020)) with one of them only achieving a maximum systolic pressure range of 140–150 mmHg. This does not model the hypertensive cat well, since a cat may be presented with a systolic BP of > 180 mmHg (Brown et al., 2007; Jepson, 2011). There can also be difficulty with non-invasive measurements in the hypotensive state, which is equally important to capture. Hypotension is most likely to occur under anaesthesia so assessing accuracy in anaesthetised cats is appropriate, but awake cats can still be at risk of this for other reasons, such as sepsis (Troia et al., 2019). Two studies (Cerejo et al., 2017; Caulkett et al., 1998) found the oscillometric device failed more frequently at lower invasive BP ranges. This reduces the number of valid measurements available for statistical analysis, and/or takes more time to obtain an answer. It is clinically important to recognise hypotension in the anaesthetised veterinary patient to correct it quickly, so it is equally important to be able to validate a device for use in this situation.

Replicating the methods of these studies in a population of known hypertensive cats in the conscious state would be informative but have numerous practical barriers. Inducing hypertensive diseases artificially in laboratory cats is challenging and may take significant time, but obtaining owner consent to perform anaesthesia and telemetry in pets where there are risks involved is equally difficult. Acclimatisation to the measurement to avoid white coat syndrome (situational hypertension due to handling stress) and management of comorbidities associated with hypertension for the duration of the study would also have to be considered. This could theoretically be mitigated in part by selecting cats surrendered to charities for rehoming, but this also raises ethical questions, and rescue centres may not be willing to engage with this.

The Bland-Altman method of analysis is the most appropriate for comparing two methods of quantitative measurement (Giavarina, 2015). For multiple methods in the same individual over time, the method should be modified (Bland & Altman, 2007). Only De Lombaert et al. (2023) did not use this method as the main aim of the study was not to evaluate accuracy.

Only four of the papers stated that the person collecting the measurements for the test method was blinded to the reference method (Cerejo et al. 2020; da Cunha et al., 2014; Binns et al., 1995; Caulkett et al., 1998), usually by having a separate person doing each. However, blinding is considered less important with an objective measurement such as blood pressure (BMJ Best Practice, 2023) than for studies requiring subjective interpretation, and it would not be possible to blind the interpretation of the data since it involves directly comparing the test and reference method. Only one paper using the Doppler method (Anjos et al., 2014) described that the person performing the measurements was experienced in the method and had received training. This could affect the results and interpretation since experience level has been shown to affect the coefficient of variability between measurements (Gouni et al., 2014), whereas oscillometry relies less on user skill or experience.

The ratio of limb/tail circumference to cuff size was shown to affect the accuracy of BP measurement (Cerejo et al., 2020) and the recommended ratio is 0.30–0.40. The range of ratios in the studies discussed was 0.30–0.60. Two studies (Martel et al., 2013; Zwijnenberg et al., 2011) did not calculate the ratio at all. Only six papers described specifically how they validated that the invasive method was accurate and reliable (Cerejo et al., 2017; Cerejo et al. 2020; da Cunha et al., 2014; Pedersen et al., 2002; Acierno et al., 2010; Cremer et al., 2020).

The sample size of five of these studies was smaller than the minimum recommended by ACVIM to validate a non-invasive device (Martel et al., 2013; De Lombaert et al., 2023; Branson et al., 1997; Pedersen et al., 2002; Zwijnenberg et al., 2011). da Cunha et al. (2014) excluded a patient from the analysis due to having outlier results, and it is not stated whether this removal affected the results, but this paper did have the largest sample size, so it is likely to be of less consequence than the other papers. Only one paper (Cremer et al., 2020) performed a power calculation, which showed the sample size was adequate to detect a significant difference between the methods.

Overall, although the study designs were individually sound and statistical analysis was suitable for the question asked, the results and conclusions from these papers are not in complete agreement. This may be due to the differences in methodology described above. However, there is more evidence for the accuracy of oscillometric measurements than for Doppler measurements. Recent work has shown that Doppler and oscillometry do not agree well with each other (Cerna et al., 2021). However, both are used widely by veterinary practices and veterinary staff may only have access to one method. In conclusion, this topic requires further research and the accuracy of non-invasive blood pressure remains a difficulty for the veterinary practitioner.

## Methodology

**Search Strategy:** 

Databases searched and dates covered:	CAB Abstracts at CABI Digital Library: 1977–2025 PubMed at National Library of Medicine: 1979–2025
Search strategy:	CAB Abstracts: (cat OR cats OR feline) (Doppler OR oscillometr*) (“blood pressure”) (invasive OR direct) 1. AND 2 AND 3 AND 4 PubMed: (cat OR cats OR feline) AND (doppler or oscillometr*) AND (“blood pressure”) AND (invasive OR direct)
Dates searches performed:	21 May 2025

**Exclusion / Inclusion Criteria:** 

Exclusion:	Species other than domestic cat, irrelevant to PICO question, not compared to invasive reference standard method.
Inclusion:	Compared to reference standard invasive telemetry, performed in domestic cats, oscillometric or Doppler measurement as test method.

**Search Outcome:** 

Database	Number of results	Excluded – irrelevant to the PICO	Excluded – not domestic cats	Excluded – no comparison to reference standard invasive method	Excluded – not available in English	Excluded – full article not available	Excluded – methodology and data not suitable for analysis	Total relevant papers
CAB Abstracts	56	33	2	9	2	0	0	10
PubMed	53	26	8	7	0	1	1	10
Citation tracking (cited by de Lombaert et al., 2023)	2	0	0	0	0	0	0	2
Total relevant papers when duplicates removed								14

## ORCiD

Fiona Douglas: https://orcid.org/0009-0002-0738-9285

## Conflict of Interest

The authors declare no conflicts of interest.

## Disclaimer

Knowledge Summaries are a peer-reviewed article type which aims to answer a clinical question based on the best available current evidence. It does not override the responsibility of the practitioner. Informed decisions should be made by considering such factors as individual clinical expertise and judgement along with patient’s circumstances and owners’ values. Knowledge Summaries are a resource to help inform and any opinions expressed within the Knowledge Summaries are the author's own and do not necessarily reflect the view of the RCVS Knowledge. Authors are responsible for the accuracy of the content. While the Editor and Publisher believe that all content herein are in accord with current recommendations and practice at the time of publication, they accept no legal responsibility for any errors or omissions, and make no warranty, express or implied, with respect to material contained within. For further information please refer to our Terms of Use.
